# Interaction of Host Cellular Proteins with Components of the Hepatitis *Delta* Virus

**DOI:** 10.3390/v2010189

**Published:** 2010-01-18

**Authors:** Valerie Greco-Stewart, Martin Pelchat

**Affiliations:** 1 Department of Biochemistry, Microbiology & Immunology, University of Ottawa, 451 Smyth Road, Ottawa, Ontario, K1H 8M5, Canada; E-Mail: vgrec079@uottawa.ca; 2 Canadian Blood Services, 1800 Alta Vista Drive, Ottawa, Ontario, K1G 4J5, Canada

**Keywords:** hepatitis *delta* virus (HDV), *delta* antigens (HDAg-S and HDAg-L), DNA-dependant RNA polymerase (RNAP)

## Abstract

The hepatitis *delta* virus (HDV) is the smallest known RNA pathogen capable of propagation in the human host and causes substantial global morbidity and mortality. Due to its small size and limited protein coding capacity, HDV is exquisitely reliant upon host cellular proteins to facilitate its transcription and replication. Remarkably, HDV does not encode an RNA-dependent RNA polymerase which is traditionally required to catalyze RNA-templated RNA synthesis. Furthermore, HDV lacks enzymes responsible for post-transcriptional and -translational modification, processes which are integral to the HDV life cycle. This review summarizes the known HDV-interacting proteins and discusses their significance in HDV biology.

## 1. Introduction

**Figure 1.**
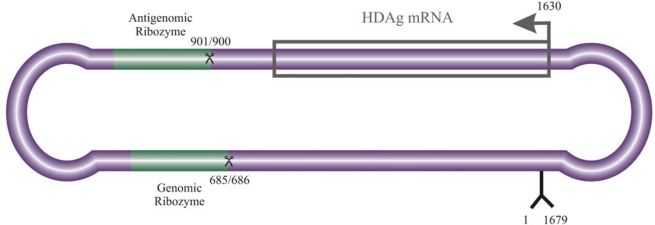
Hepatitis *delta* virus RNA. The hepatitis *delta* genome is depicted with the superimposition of both genomic and antigenomic polarities. The *delta* ribozyme motifs are highlighted and cleavage sites indicated. A putative promoter on the genomic strand and region encoding the ∼800 nt HDAg mRNA are also shown. Numbering is in accordance with [9].

The hepatitis *delta* virus (HDV) is the smallest known mammalian pathogen and is responsible for exacerbation of hepatitis disease progression upon co-infection with the hepatitis B virus (HBV). The HDV genome is comprised of a small, G/C-rich, single-stranded, circular RNA molecule of ∼ 1,700 nt and adopts a highly base-paired, rod-like secondary structure (for general reviews of HDV biology and replication, see [1–5], Figure 1). It replicates in the nucleus by a symmetrical, rolling circle mechanism [6,7] in which a multimeric genomic species is synthesized, cleaved to unit length monomers by endogenous *delta* ribozymes, and ligated by host proteins [8]. The process is repeated to generate the antigenomic species. The genome is the most abundant HDV species and accumulates to levels in excess of 10 times that of the antigenome [1].

**Figure 2.**
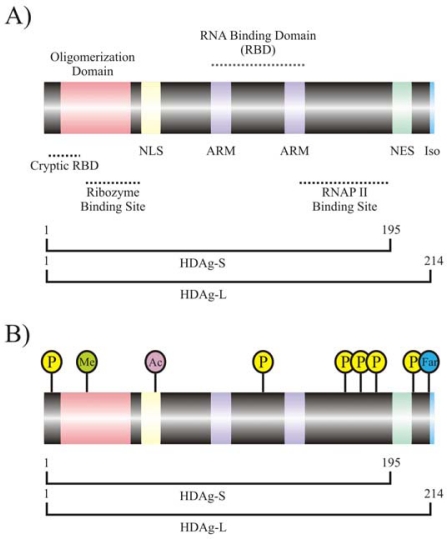
Schematic representation of HDAg. **A)** Representation of HDAg showing significant functional regions. HDAg encompasses amino acids 1 to 195; the C-terminal expansion of HDAg-L (amino acids 196 to 214) is also indicated. NLS: nuclear localization signal (amino acids 66–75); NES: nuclear export signal (amino acids 198–210); ARM: arginine-rich motifs (amino acids 97–107 and 136–146); Iso: isoprenylation signal for the addition of farnesyl (amino acids 211–214). The cryptic RNA-binding domain (RBD) spans amino acids 2–27 while the main RBD spans both ARMs. The coiled-coil oligomerization domain spans amino acids 12–60. Ribozyme binding occurs at amino acids 24–50 and the RNAP II binding is localized to the C terminus at amino acids 150–195. **B)** Modification of HDAg. All the known naturally-occuring modifications are shown concurrently, though not every modification is present simultaneously *in vivo*. Lettering for the modifications is P: phosphorylation, Me: methylation, Ac: acetylation, Far: farnesylation. Sumoylation of multiple lysine residues of HDAg-S has also been reported.

HDV contains one open reading frame encoding the 24 kDa small *delta* antigen (HDAg-S; 195 aa; [10]), which is believed to play roles in transcriptional initiation and elongation during HDV infection (Figure 2) [11,12]. Post-transcriptional editing of HDV antigenomic RNA results in transcriptional readthrough and production of the 27 kDa large *delta* antigen (HDAg-L; 214 aa; Figure 2) [13–15]. The C-terminal expansion of HDAg-L confers functional properties to this protein that differ from HDAg-S; the additional 19 amino acids at the C-terminal domain of HDAg-L serve as an assembly signal for HDV virion formation [16], and is thus necessary for viral packaging [17–19], a process that involves encapsidation with HBV envelop proteins (reviewed by [20]). HDAg-L also acts as a negative regulator of HDV transcription late in the HDV life cycle [21]. Remarkably, HDV lacks an RNA-dependent RNA polymerase, which would speculatively be required for its replication. This observation has inspired a large body of investigation into the mechanisms by which HDV replicates in terms of host protein utilization throughout the HDV life cycle, particularly with respect to transcription and replication.

## 2. Interaction of host cellular proteins with HDV RNA and HDAgs

All viruses must subvert host cellular machinery for genome expression and virion production. HDV is almost completely reliant upon the host cell for these processes due to its extremely limited protein coding capacity. Several host proteins interact with HDV during the course of infection, including those in the nucleus that facilitate transcription and replication and those in the cytoplasm that are principally associated with production of HDAgs. Host proteins that interact with components of HDV can be divided into two categories – those that interact with HDAg (Table 1) and those that interact with HDV RNA (Table 2). While the biological significance of some of these intermolecular interactions is known, the role of most of these interactions in the life cycle of HDV remains unknown. The following sections will discuss the current body of knowledge with respect to two types of HDV-mediated host-pathogen interactions: those occurring between host proteins and HDAg and those occurring between host proteins and HDV RNA.

## 2.1. Interaction of HDAgs with host proteins

Co-immunoprecipitation studies of cells expressing HDAg-S have recently reported that over 100 cellular proteins might interact with HDAg-S alone; over 25 percent of these proteins were shown to affect HDV RNA accumulation [40]. These HDAg-S-interacting partners included RNA polymerase subunits, heterogeneous ribonucleoproteins (hnRNPs), helicases, RNA-binding proteins, and transcription and splicing factors [40]. Although the precise role of these interactions in the HDV life cycle is unclear, it is evident that HDAgs interacts with numerous host proteins during the course of infection. However, roles have been suggested for some of the elucidated HDAg-interacting proteins; these proteins and their putative functions with respect to HDV biology will be discussed in the present section.

Cellular proteins involved in post-translational modification have been shown to play a critical role in HDV biology. HDAgs undergo much post-translational modification including serine and threonine phosphorylation [46–48], arginine methylation [29], lysine acetylation [31], and cysteine farnesylation [25,26]. These modifications contribute to processes such as subcellular localization and efficiency of HDV RNA synthesis, possibly by regulating intermolecular interactions involving HDAgs [29,31,48]. It has recently been proposed that the modifications present on HDAg-S might dictate which RNA species, such as genomic, antigenomic, or HDAg mRNA, is produced during HDV RNA synthesis [24,49]. Post-translational modifications are performed by numerous cellular proteins, many of which have been identified (Table 1).

Two phosphorylated isoforms of HDAg-S and one phosphorylated isoform of HDAg-L are found in infected cells [47]. While HDAg-S is phosphorylated on both serine and threonine residues, HDAg-L is phosphorylated only on serine residues [47]. Multiple kinases have been associated with HDAg phosphorylation including casein kinase II (CKII; [22]), protein kinase C (PKC; [22]), double-stranded RNA-activated protein kinase R (PKR; [23]), and extracellular signal-related kinases 1 and 2 (ERK1/2; [24]). Phosphorylated residues show a high level of conservation among HDV isolates, and different phosphorylation states of HDAg have been associated with transcriptional enhancement, specifically in the case of genomic RNA synthesis [22,24,48,50]. Protein arginine methyltransferase 1 (PRMT1) methylates arginine residues located in the glycine- and arginine- rich (GAR) region of the putative, cryptic RNA-binding domain of HDAg, and it has been shown that replication of HDV RNA, specifically the genomic species, requires methylation of arginine 13 of HDAg [29]. It has also been shown that HDAg methylation promotes nuclear retention of HDAg, which might be the reason for the observed transcriptional enhancement. Lysine acetylation, performed by the histone acetyltransferase (HAT) domain of p300 cellular acetyltransferase, has also been shown to promote nuclear retention of HDAg and increase transcription of the three major HDV RNA species [30,31]. Acetylation of lysine 72 specifically promotes an increase in early HDAg-L synthesis, possibly mediating the transition from active RNA synthesis early in the life cycle of HDV to moderate synthesis in preparation for viral export later during infection [31]. Recently, it has been shown that SUMO1, the small ubliquitin-related modifier isoform 1, and Ubc9, a SUMO conjugating enzyme, interact with HDAg-S causing sumoylation of multiple lysine residues [32,51–53]. SUMO modification is a reversible post-translational process in which a ubiquitin-like protein is conjugated to target lysine residues; viral proteins are typically modified with by the SUMO1 isoform [52]. As observed with other post-translational modifications, HDV genome and HDAg mRNA synthesis are enhanced by sumoylation of HDAg-S, further supporting a role of post-translational modification of HDAg in regulation of HDV RNA synthesis [32]. Finally, The 19 amino acid expansion of the carboxy terminal domain (CTD) of HDAg-L contains the isoprenylation signal CRPQ and is isoprenylated with farnesyl at cysteine 211 [25,26]. Farnesylation causes a conformational change in HDAg-L which masks 32 amino acids at the CTD of the protein, possibly explaining its different functions as compared to those of HDAg-S [27]. This modification is suggested to be performed by protein farnesyltransferase (FTase), and the farnesylated form of HDAg-L causes *trans*-repression of HDV transcription in the nucleus and facilitates viral packaging with HBV envelope proteins [27,28]. HDAg modification is thus believed to promote subcellular localization of HDV ribonucleoprotein (RNP) particles and enhance synthesis of specific HDV species at precise times during the HDV life cycle.

Cellular proteins involved in post-transcriptional modification thus play a crucial role in progression of the HDV life cycle through modulation of HDAg function. Despite the propensity for extensive post-translational modification of HDAg, cellular HDAg exists in several states of post-translational modification in infected cells and it is unclear which proportion of HDAg is modified, and the nature and extent of these modifications, during infection.

In addition to several sites for post-translational modification, HDAg contains a nuclear localization signal (NLS) composed of ten amino acids located at position 66–75 that is necessary for nuclear entry of HDV RNPs [54]. This sequence represents a classical type 1 NLS, which is characterized by a large proportion of basic amino acids [55]. The arginine-rich RNA binding motifs and NLS of HDAg are required for nuclear entry of HDV RNA [56]. Nuclear entry of HDV RNPs is facilitated by karyopherin (importin) 2α, part of the nuclear pore complex that specifically binds to canonical NLS motifs to regulate entry of cytoplasmic components into the nucleus [33]. Following nuclear entry and replication, HDV RNA, in the form of RNPs, must be exported from the nucleus for virion packaging and cellular export. HDAg-L contains a nuclear export signal (NES) spanning residues 198–210 [57]. Specifically, proline 205 plays an essential role in HDV RNP trafficking out from the nucleus [57]. Recently, a novel protein, the nuclear export signal-interacting protein (NESI), was identified as an HDAg-L-interacting partner [34]. NESI is predominantly expressed in the liver and, when NESI expression is inhibited, HDV RNA fails to accumulate in the cytoplasm of infected cells and packaging of HDV RNPs is prevented. The extended C-terminal terminal domain of HDAg-L also contains a clathrin adaptor homology region, which might facilitate clathrin-mediated exocytosis [35,36]. Specifically, HDAg-L interacts with the clathrin heavy chain, and this interaction is required for HDV virion assembly [35]. The clathrin-binding activity of HDAg-L is conserved across HDV genotypes, suggesting that interference with clathrin-mediated endo- and exocytosis during HDV particle maturation might be implicated in HDV disease progression [35,36]. These data confirm a unique and specific role for HDAg-L in viral export and packaging and provide evidence of host cell interaction with HDAg during the HDV life cycle. From these data, it can be ascertained that HDV undergoes conventional interactions with cellular proteins that mediate cellular trafficking in order to enter specific subcellular domains to achieve replication and virion assembly.

Nuclear proteins provide attractive candidates for HDAg-interacting proteins that regulate transcription and replication. Nucleolin, also known as C23, is a multifunctional nuclear phosphoprotein that has been demonstrated to interact with HDAg [37]. Nucleolin is localized to the nucleolus and plays numerous roles including synthesis and maturation of the ribosome, cell proliferation and growth, nuclear trafficking, cytokinesis, nucleogenesis, transcriptional repression, replication, signal transduction, and chromosome remodeling (for a review, see [68]). Although it has many properties including DNA-helicase and DNA-dependent ATPase activity, nucleolin also possesses helicase IV activity and can unwind RNA helices [68,69]. Both HDAg-S and HDAg-L interact with nucleolin in cells, and accumulation of the antigenome increases concurrently with levels of nucleolin, implicating a role for this protein in HDV replication [37]. The nucleolin-binding domain of HDAg resides in the N-terminus in a lysine/arginine-rich region, which is conserved among HDV isolates, and it was shown that this domain is necessary for nucleolar targeting and accumulation of HDV RNAs. However, more recent studies have suggested that the nucleolin-HDAg interaction might be mediated by binding of rRNA precursors; both proteins contain RNA-binding domains (RBDs) capable of rRNA interaction [70,71]. Furthermore, co-localization of HDAg with nucleolin in the nucleolus occurs in the absence of active HDV RNA synthesis and accumulation [70]. Additional clarification of the precise role of nucleolin in HDV RNA synthesis is thus required.

Nucleophosmin, also known as B23, is another nuclear phosphoprotein that has been shown to interact with both HDAg-S and HDAg-L [38]. Like nucleolin, nucleophosmin has myriad functions including roles in cell growth and proliferation, nuclear shuttling, and ribosome biogenesis (reviewed by [72]). Although predominantly associated with the nucleolus, nucleophosmin binds to the NLS of proteins to facilitate shuttling between the nucleus and the cytoplasm; it has recently been shown to mediate nuclear import of the viral protein Rev from HIV-1 [73,74]. Cells replicating HDV and cells expressing HDAg were both shown to upregulate nucleophosmin mRNA transcription, and nucleophosmin was shown to co-localize with HDAg in the nucleolus [38]. Binding of HDAgs to nucleophosmin occurs through the N-terminal of HDAg in a region encompassing the NLS but not the coiled-coil domain necessary for HDAg oligomerization. Furthermore, HDAg exists in a large complex with both nucleophosmin and nucleolin, and increasing the amount of nucleophosmin is associated with an increase in the amount of HDV RNA present in cells. From these data, it would appear that nuclear phosphoproteins such as nucleophosmin and nucleolin form RNP complexes within the nucleoli of infected cells to localize HDV RNPs to potentially enhance HDV replication.

Yin Yang 1 (YY1) is a transcription factor that binds both nucleophosmin and nucleolin and has recently been shown to interact with both HDAg-S and HDAg-L [30]. Through interaction with numerous other nuclear and cytoplasmic proteins, YY1 can activate and repress gene expression involved in many cellular functions such as embryogenesis, differentiation, and proliferation (reviewed by [75]). The central domain of HDAg containing its principal RBD interacts with the zinc-finger region of YY1 in a manner which is independent of HDV RNA, and these proteins, as well as CBP, and p300 acetyltransferases, are found in large complexes [30]. A role for YY1 in HDV replication is presumed based on the previously reported putative functions nucleolin and nucleophosmin, and it was observed that levels of both genomic and antigenomic HDV transcripts increase in the presence of increasing amounts of YY1. An increase in YY1-associated acetyltransferases also increases HDV replication; acetyltransferase activity is specifically required for enhancement of HDV RNA accumulation. These data suggest that many nuclear and nucleolar proteins that exist in large RNP complexes could play a role in HDV RNA accumulation and/or synthesis, although the precise role of proteins such as nucleolin, nucleophosmin, and YY1 in HDV biology has not yet been conclusively determined.

Histone H1e is another nuclear protein that was identified as an HDAg-S interacting partner [42]. Histones are proteins involved in DNA condensation and organization into chromatin [76,77]. In the nucleus, DNA winds around histone octomers to form core particles, which are connected by H1 linker proteins to form nucleosomes, the fundamental units of chromatin architecture (reviewed by [78]). Linker histones are among the most abundant nuclear proteins and have been implicated in both the organization of higher chromatin structure and the modulation of gene expression by controlling the access of regulatory proteins to their DNA targets. The H1 family is contains eleven variants; the H1e variant, also known as histone H1.4, is ubiquitously and constitutively expressed in somatic cells. HDAg-S interacts with histone H1e through its leucine zipper motif, while HDAg-L is unable to undergo this interaction due to conformational masking of this region because of determinants located upstream of the isoprenylation signal [42]. It is believed that histone H1e assists HDV replication based on a reduction in HDV RNAs in cells harboring H1e mutants defective in HDAg-S binding; this reduction is reversible by complementation with wild-type H1e [42]. These observations support the theory that H1e might play a role in HDV replication and transcription by a yet unknown mechanism.

The protein MOV10 was recently shown to interact with HDAg [43]. Interestingly, the plant homologue of MOV10 is a putative RNA helicase that associates with Argonaute proteins to facilitate RNA interference [79]. When MOV10 expression is prevented, HDV replication is inhibited [43]. Inhibition occurs at the level of transcription and not HDAg translation, and the presence of Argonaute-4 protein is also required for HDV transcription. The authors hypothesize that these components, both downstream effectors in the microRNA (miRNA) interference system, could function by remodeling HDV RNA upon cell entry to facilitate transcription initiation competency. These data suggest that HDV RNA could be involved in complex RNA regulatory pathways in host cells, which are only now being characterized.

A potential human homologue of HDAg-L known as *delta* interacting protein A (DIPA) has also been identified [41]. DIPA shares 24% amino acid identity and 56% sequence similarity with HDAg-L, although whether or not an ancestral relationship exists between DIPA and HDAg is unclear. DIPA interacts with various nuclear proteins to repress gene expression, acting as a transcriptional regulator *in vivo* [80,81]. Overexpression of DIPA in cells replicating HDV has been shown to result in attenuation of HDV replication, suggesting that it might serve a similar function in HDV RNA synthesis as HDAg-L, although a definitive role for DIPA in HDV biology has not been established [41]. Furthermore, whether or not DIPA constitutes a true HDAg-L homologue remains unclear since additional bioinformatic analyses have disputed the true level of homology between these two proteins [82]. Further studies will thus be required to determine if DIPA has any function in the HDV life cycle.

While many proteins have been shown to interact with HDAgs, the role of most of these proteins in the HDV life cycle and their relation to HDV-mediated pathogenesis remains unclear. One possible mechanism by which HDV coerces host cell replication and virion assembly is through host cell gene regulation. The following section will discuss HDAg interactions with host cell components that results in alteration in gene expression.

### 2.2. HDAg interaction with host transcription factors: A possible role for HDV in host gene regulation

Genome-wide proteomic analyses of Huh7 hepatocellular carcinoma cells transfected with HDV RNA, HDAg-S, or HDAg-L have shown that at least 32 proteins demonstrate differential expression in the presence of HDV components [83]. The affected proteins are involved in diverse processes such as nucleic acid metabolism, protein metabolism, cellular transport, signal transduction, apoptosis, and cell growth. This altered global protein expression profile could provide insight into the cellular factors with which HDV components interact in infected cells and suggest mechanisms of HDV pathogenesis. However, the manner in which HDAgs regulate host cell gene expression at various stages of the HDV life cycle remains poorly understood.

HDAg, particularly HDAg-L, has been shown to regulate gene expression through a number of cellular pathways. RNAP II transcription from both active and basal promoters has been shown to be inhibited by both isoforms of HDAg *in vitro* and *in vivo*, presumably by an indirect method such as association with or sequestration of host transcription factors [84]. However, later studies would demonstrate that HDAg-L is predominantly responsible for activating transcription from numerous types of bacterial [41] and eukaryotic promoters [85] in various cell lines in a manner that is independent of farnesylation. However, the mechanism of this HDAg-L-mediated gene activation and repression remains unclear.

More recently, HDAg-L has been demonstrated to stimulate transcription from the serum response element (SRE) pathway in a manner that does not change the DNA-binding activity of the serum response factor (SRF) protein, the endogenous transcription factor associated with SRE-responsive gene regulation [86,87]. It has also been shown that MxA, an interferon- α (IFN- α)-inducible mediator of the cellular antiviral response, can be activated by HDAg-L [88]. Although MxA activation is suspected occur through HDAg-L interaction with the transcription factor STAT-3, direct interaction between these two proteins has not been demonstrated despite their association in large cellular protein complexes. HDAg-L, and to a lesser extent HDAg-S, have also been shown to repress HBV gene expression [85,88,89]. Such regulation of host and HBV transcription might provide an optimal intracellular environment for HDV propagation.

To date, there are few documented instances of direct HDAg interaction with cellular transcription factors resulting in altered host gene expression. In one case, HDAg-S and HDAg-L have been shown to interact with the Smad3 and c-Jun transcription factors both *in vitro* and *in vivo* [44]. Smad3 is a component of the transforming growth factor-β (TGF- β) signal transduction pathway which can activate or repress gene expression following its activation and nuclear translocation [90]. c-Jun is a member of the activator protein-1 (AP-1) family of transcription factors which regulate gene expression in response to numerous stimuli [91]. While interaction of HDAg with both transcription factors is mediated by determinants located in the N-terminal domain of HDAg, only HDAg-L stimulates expression of TGF- β- and AP-1-responsive genes [44]. Both HDAgs perpetuate TGF- β pathway signaling by inhibiting c-Jun-mediated repression of Smad3-regulated genes. Farnesylation of HDAg-L is essential for these processes, which might account for the differential abilities of the HDAgs to stimulate the TGF- β pathway. Additional studies have identified the TRAF2 signal transducing protein as an HDAg-interacting partner that stimulates tumor necrosis factor (TNF)-α-induced NF-κB signaling [45]. Although both HDAg isoforms interact with TRAF2, only HDAg-L activates this pathway and farnesylation of the CTD of HDAg-L is not required for this type of gene activation [45]. From these observations, it is suggested that alteration of gene expression in cells by HDAgs could account for the cytopathic effects observed during HDV infection due to upregulation of cytokines, inflammatory enzymes, growth factors, and anti-apoptotic proteins [45] and compounds that induce hepatic fibrinogensis [44].

Clustrin, a secreted protein with chaperone activity that is involved in the regulation of cell death (reviewed by [92]), has been recently been demonstrated to be upregulated in the presence of HDAgs [93]. Both isoforms of HDAg as well as HDV replication are associated with enhanced clustrin expression. Acetylation of histone H3 induces clustrin expression, and it was shown that cells replicating HDV exhibit an increased amount of H3 acetlyation and clustrin expression, particularly in the presence of HDAg-L later in the HDV life cycle. These data thus suggest that HDAg epigenetically induces clustrin expression through enhancement of histone H3 acetylation, although no direct interaction between HDAg and histone H3 has been demonstrated. Since clustrin expression is enhanced in some cancers, it is possible that this proposed form of HDV-mediated dysregulation of human genes might contribute to pathologies such as hepatocellular carcinoma that are observed in HDV patients. Histone acetylation is thus one plausible mechanism of HDAg-mediated host gene regulation based on these observations, the previously elucidated interaction between HDAg and histone H1e1 [42], and HDAg association with complexes containing histone acetyltransferases [30].

In the absence of direct interaction with host transcription factors, little is known about the additional ways in which HDV infection might regulate host gene expression. It has been shown that the RNA binding domain of HDAg bears considerable homology to canonical DNA-binding domains (DBDs) such as the one found in the eukaryotic SRY gene [82]. This observation suggests that HDAg might be capable of direct interaction with cellular DNA promoters to stimulate gene activation or repression. As aforementioned, HDAgs also interact directly with numerous nuclear factors that are associated with transcription such as nucleolin [37], nucleophosmin [38], YY1 [30], and DIPA [41]; though studies have not demonstrated the effect of these interactions on gene expression, the possibility exists that HDAg might exert its role in genetic regulation through interaction with such proteins. Since HDAg-L is the isoform that is predominantly associated with gene activation and repression, it is possible that HDAg-S might indirectly regulate gene expression through interaction with transcription factors while HDAg-L is capable of direct *trans*-activation of host genes, providing a temporal regulation of host gene expression dependent on the stage of HDV infection. Further studies will be required to fully elucidate HDAg interacting proteins and determine their involvement in host cell gene regulation.

### 2.3. HDV interacts with eukaryotic RNAPs for transcription and replication

One of the most important aspects of HDV biology is that HDV must interact with and utilize host DNA-dependent RNA polymerases (RNAPs) for transcription and replication since it does not encode an RNA-dependent RNA polymerase. Since HDAg-S was shown to be necessary for the accumulation of HDV RNA in infected cells [11], it was speculated that HDAg-S must interact with at least one host cellular polymerase. Several lines of evidence have implicated RNAP II, and to a lesser extent RNAP I, in HDV RNA synthesis through interaction of HDAgs and HDV RNA with components of host polymerase complexes. Sensitivity of HDV RNA synthesis to α-amanitin [94,95], post-translational modification of HDAg mRNA with a 5′ methylguanine cap [96] and poly(A) tail [97,98], co-localization with RNAP II-containing transcriptionally-active SC35 nuclear subdomains [99], and *in vitro* transcription assays [65,100,101] all support a role for RNAP II in HDV transcription.

HDAg bears weak homology to the A polypeptide chain of negative elongation factor (NELF), a transcriptional regulatory factor that interacts directly with RNAP II to repress transcription [12]. NELF and DRB sensitivity-inducing factor (DSIF) repress transcriptional elongation; both HDAgs were shown to interact directly with DSIF to relieve DSIF/NELF-mediated transcriptional repression and stimulate transcriptional elongation *in vitro* [12]. HDAgs, particularly HDAg-S reverses this repression and stimulates elongation in the absence of DSIF/NELF. These observations describe a mechanism of action for HDAg-S-mediated enhancement of HDV RNA synthesis by RNAP II, and suggest a possibility for direct interaction between HDAgs and RNAP II.

Subsequent studies would indeed demonstrate the direct interaction of HDAg with RNAP II during RNAP II transcription [12,39]. HDAg-S can bind both the hypo- and hyperphosphorylated forms of RNAP II found during transcriptional initiation and elongation, respectively. Specifically, HDAg-S binds the mobile clamp element of RNAP II and makes direct contact with the Rpb1 and Rpb2 subunits [39], consistent with recent *in vivo* co-immunoprecipitation experiments [40]. The HDAg-S-RNAP II interaction is dependent on elements contained in the CTD of RNAP II [39] and phosphorylation of the conserved S177 residue [24,39]. It has recently been reported that phosphorylation of S177 of HDAg-S dictates specific interaction with the hyperphosphorylated form of RNAP II, whereas HDAg-S bearing unphosphorylated S177 interacts with the hypophosphorylated polymerase, potentially regulating which HDV RNA species are synthesized [102]. HDAg-S also accelerates forward translocation of the polymerase complex; this presumably occurs by loosening the clamp module, causing a concurrent loss of transcriptional fidelity [39,103]. Since binding of HDAg-S affects the recognition of incoming nucleotides and influences the choice of nucleotide incorporated into the transcript [39], it is possible that template recognition (*i.e.,* RNA *versus* DNA) is also affected, providing a mechanism by which RNAP II is capable of utilizing this HDV RNA template.

HDV RNA has also been shown to interact directly with RNAP II and its components. Ribonucleoprotein immunoprecipitation assays have shown interaction between both genomic and antigenomic HDV RNA with RNAP II in cells replicating HDV [63,64]. Interaction with TATA-binding protein, a component of the RNAP II general transcription factor TFIID, has also been demonstrated in this manner [62]. Direct binding of RNAP II and TBP to a segment of RNA derived from the right-terminal stem-loop domain of the HDV genome has also been demonstrated *in vitro* [65]. This RNA species has been previously been reported to contain a promoter for HDV RNA synthesis [100]. The structure of purified RNAP II engaged in transcription from an RNA species derived from the extreme tip of the left terminal stem-loop domain of the HDV antigenome, a region containing another putative HDV RNA promoter [101], has also been reported [66]. Together, these observations support a role for RNAP II in HDV RNA synthesis, but do not exclude other polymerase involvement in HDV biology.

The observation that replication of HDV, under some circumstances, is resistant to high levels of α-amanitin has prompted investigation into the involvement of RNAP I in HDV replication [7,104]. Metabolic labeling experiments have demonstrated that the genomic and antigenomic strands of HDV RNA are transcribed in separate nuclear compartments: antigenome synthesis takes place in the nucleolus and is largely resistant to inhibition by to α-amanitin, whereas genome synthesis occurs throughout the nucleoplasm and is sensitive to α-amanitin [105]. These observations suggest that synthesis of different HDV RNA species might occur via different host polymerases. HDAg-S was specifically shown to co-immunoprecipitate with SL1, a component of the RNAP I transcriptional machinery, and *in vitro* synthesis of the antigenome is largely diminished upon immunodepletion with α-SL1 antibody, implicating RNAP I in antigenome synthesis [105]. However, additional studies have failed to demonstrate interaction between RNAP I and HDAg-S by co-immunoprecipitation, allowing for the possibility that direct interaction of HDAg-S with RNAP I might not occur during RNAP I-mediated synthesis of HDV RNA [106]. Interestingly, post-translational modifications of HDAg, including methylation of R13, acetylation of K72, and phosphorylation of S177 have differing effects on synthesis of the three HDV-derived transcripts; synthesis of the genome and HDAg mRNA requires these modifications while they are unnecessary for antigenome synthesis [24]. These studies thus propose that post-translational modification of HDAg might serve as a molecular switch for the determination of which HDV RNA species is synthesized during a particular stage of HDV infection.

Both genomic and antigenomic HDV RNA were recently shown to associate with RNAP I in cells replicating HDV [62]. Interestingly, RNAP III was shown to undergo similar interactions, though the physiological significance of these associations remains unclear. It is possible that HDV RNA binds common polymerase subunits or shared transcription factors such as TBP [62,65] as has been demonstrated for HDAg in binding of the shared Rpb8 polymerase subunit [40]. Whether or not these associations are required in HDV replication is uncertain. However, these observations do prompt further investigation into the role of various host polymerases in HDV replication.

### 2.4. Interaction of HDV RNA with host proteins

HDAg itself is an RNA binding protein; HDAg interacts with HDV RNA and is proposed to shuttle it to the nucleus for replication, to the cytosol for packaging and export, and to modulate its transcription and replication (reviewed by [2,3]). HDAg was first identified as an RNA-binding protein through demonstration of its ability to interact with genomic HDV RNA [46], and it has subsequently been shown that both polarities of HDV RNA interact with HDAg in cells replicating HDV [64,107]. The N-terminal two-thirds of HDAg are extremely basic and contain two stretches of amino acids with similarity to the leucine zipper motif characteristic of nucleic acid-binding proteins, and it was demonstrated that HDAg binds to HDV-derived RNAs through this region [108]. The HDAg RNA-binding region was further localized to two arginine-rich motifs (ARMs) separated by 29 amino acids within the central region of HDAg; both ARMs, specifically the basic amino acids contained therein, are necessary for the RNA-binding activity of HDAg [109]. Together, the ARMs constitute the principal RBD of HDAg. Binding of HDV RNA to the ARM regions appears to enhance HDV replication *in vivo* since RNA accumulation is impaired by deletion of these motifs [109]. Further studies of HDAg revealed a cryptic RBD located at the extreme N-terminal domain of HDAg between residues 2 and 27 [110]. This RBD is capable of binding both the genome and antigenome and was found to play a role in the ability of HDAg to act as a chaperone to modulate the activity of the *delta* ribozyme [111]. The binding activity of HDAg to HDV RNA is necessary for RNP particle formation during transcription and virion packaging [112]. Multimerization of HDAg has also been shown to be necessary for efficient and HDV RNA binding and proper HDV RNP formation [113]. The central, ARM-containing region of HDAg demonstrates a high level of conservation among HDV isolates [114], possibly due to its role in the HDV RNA binding which is necessary for HDV virion assembly.

The unbranched, rod-like secondary structure of HDV RNA is necessary for HDAg binding [108,115]. This secondary structure is attractive to RNA-binding proteins, and several host proteins have been demonstrated to bind, and in some cases modify, HDV RNA. As aforementioned, modification of the HDV antigenome in the region encoding the HDAg-S mRNA is responsible for the production of HDAg-L. The UAG amber termination codon of HDAg-S is converted to UIG, where inosine is read as guanine, yielding the codon for tryptophan. Since modification occurs on the antigenome, or on an RNA that can be processed into the antigenome, this nucleotide conversion is retained in subsequent rounds of replication and is incorporated into the HDAg-S mRNA, resulting in translational readthrough which generates HDAg-L [13,116]. This conversion of adenosine to inosine is carried out by a double-stranded RNA adenosine deaminase [14,116]. Specifically, the small isoform of adenosine deaminase acting on RNA (ADAR 1) has been shown to edit the HDV RNA antigenome [15]. HDAg-S can repress this editing event, subsequently delaying the production of HDAg-L and regulating the stages of the HDV life cycle [117]. ADAR 1 is thus an essential host protein necessary for the progression of HDV infection.

Another critical step in the replication of HDV is the autocatalytic cleavage of its RNA genome and antigenome by the *delta* ribozyme. It was reported that glyceraldehyde 3-phosphate dehydrogenase (GAPDH) interacts with the antigenome of HDV to enhance *delta* ribozyme activity [58]. Interaction of GAPDH with the genomic strand of HDV RNA was also recently demonstrated [59]. Although conventionally attributed to having a metabolic role in glycolysis, GAPDH has also been shown to exhibit nuclear translocation under specific circumstances such as drug treatment, hyperglycemic stress, cell entry into S-phase, and viral infection where it can be found in association with complexes involved in such diverse functions as apoptotic cell death, DNA proofreading, nuclear fusion, and telomere maintenance [118]. GAPDH plays a role in transcriptional activation of histone H2B, indicating that this protein might be involved in RNA regulation [119]. The GAPDH-HDV interaction enables the shuttling of GAPDH to the nucleus and enhances *delta* ribozyme activity almost two-fold in agreement with a previous study, which showed that GAPDH increases the rate of *cis*-cleavage of hammerhead ribozymes [58,120]. These observations suggest that GAPDH enhances HDV replication by acting as a molecular chaperone that facilitates the unwinding of HDV RNA to enable autocatalytic cleavage during rolling circle replication.

As aforementioned, PKR is a protein that interacts with and phosphorylates HDAg [23]. It has also been shown that the genome, antigenome, and subgenomic fragments of HDV RNA bind and activate PKR [60,61]. This observation is unusual since PKR activation typically occurs in the presence of extensively base-paired, double-stranded RNA (dsRNA); binding of highly-structured, single-stranded RNA (ssRNA) such as viral RNA usually inhibits PKR activity [121]. When activated, PKR mediates the interferon-induced antiviral response and acts as a tumor suppressor by inhibiting cellular translation; paradoxically, while HDV RNA activates PKR, it fails to inhibit protein synthesis *in vitro* [61,121]. Binding of specific viral ssRNA structures suppresses PKR activation to facilitate viral propagation, so it is thought that HDV interaction with PKR might serve this purpose despite this non-conventional method of translational inhibition [61]. It is possible that interaction of HDV RNA with PKR mediates its recruitment to RNP complexes containing HDAg so that PKR can phosphorylate HDAg, although this theory has yet to be tested [23,61]. Further studies of this interaction will be required to determine the precise role of PKR in the HDV life cycle.

Recently, five additional proteins, eEF1A1, ASF/SF2, hnRNP-L, PSF, and p54^nrb^, were identified as HDV RNA-interacting partners both *in vitro* and in cells replicating HDV [59,67]. Due to their strong and specific interactions with both polarities of HDV RNA, it is possible that these proteins could play a role in HDV RNA synthesis. Although the translation factor eEF1A1 is primarily a cytosolic protein, it does bind highly-structured ssRNA such as aminoacyl-tRNA [122] and has been reported to be involved in viral RNA synthesis through association with viral replicases [123–126]. It has also recently been shown to act as a chaperone used to recruit HIV RNA to RNAP II using the HIV TAR element [127]. ASF/SF2 and hnRNP-L belong to families of abundant nuclear proteins associated with pre-mRNA processing that are also frequently linked to viral replication (for a review see [128]). Furthermore, hnRNP-L was recently identified as an HDAg-S interacting protein [40]. The polypyrimidine tract-binding protein (PTB)-associated splicing factor (PSF) is a multifunctional protein involved in many processes such as splicing, polyadenylation, transcriptional regulation, retention of defective RNAs, nucleic acid unwinding and annealing, nuclear shuttling, and pH homeostasis (reviewed by [129]). p54^nrb^ shares much homology with the C-terminal portion of PSF [130] and has also been demonstrated to interact with HDAg-S [40]. Both PSF and p54^nrb^ have been shown to interact with the CTD of RNAP II during both transcription initiation and elongation, and PSF can likely interact with RNA and the CTD of RNAP II simultaneously [131]. Interestingly, PSF interacts with the same regions of HDV RNA that bind RNAP II [63,67], including a portion of the right-terminal stem-loop domain of the genome reported to demonstrate promoter activity [65,100]. It was shown that the ability of RNAP II to interact with this section of HDV RNA requires both the PSF-HDV RNA interaction and the RNAP II-PSF interaction [132]. These observations suggest that PSF, alone or in a complex with p54^nrb^, might provide a direct physical link between HDV RNA and RNAP II, promoting RNAP II-mediated transcription from a non-canonical RNA template. Further characterization of the role of PSF in the HDV life cycle might thus provide novel insight into the mechanism of HDV replication by a host DNA-dependent RNA polymerase. Furthermore, exploration of the role of PSF in HDV biology could provide clues to the mechanism of HDV pathogenesis since mutations in the PSF gene have been identified in both cervical cancer and papillary renal carcinoma cell lineages [133,134] and PSF dysregulation is associated with induction of several oncogenes [135–138]. Due to the versatility and abundance of these five proteins, their abilities to interact with HDV RNA, and their previously-reported involvement in viral replication, it is tempting to speculate that these proteins interact with HDV RNA to facilitate HDV RNA synthesis, potentially through acting as transcription factors mediating the recruitment of HDV RNA to the RNAP II complex.

## 3. Concluding Remarks

The rapidly increasing number of host proteins that interact with HDAgs and HDV RNA is testament to the complexity of the host-pathogen interaction upon HDV infection. Such interactions might enable HDV propagation, exert pathogenic effects on the host, or be completely benign; it is thus imperative to determine which host cellular proteins interact with HDV following infection and to establish their roles in the HDV life cycle and pathogenesis. Through identification of HDV-interacting factors and determination of their roles in HDV biology it will become possible to understand the molecular mechanisms by which HDV causes disease and to develop novel strategies to combat HDV infection. More generally, characterization of the manner in which HDV RNA is capable of replication and transcription by using host proteins, specifically DNA-dependent RNA polymerases, will provide global insight into polymerase function and potentially reveal novel pathways of RNA synthesis, metabolism, and regulation.

## Figures and Tables

**Table 1.** Host proteins that interact with HDAg.

**Host protein**	**Proposed Function**	**References**
	**Post translational modification**	
Casein Kinase II (CKII)	Phosphorylation (S2 and S213)	[22]
Double-stranded RNA-activated protein kinase R (PKR)	Phosphorylation (S177, S180, T182)	[23]
Extracellular signal-related kinases 1 and 2 (ERK1/2)	Phosphorylation (S177)	[24]
Protein Kinase C (PKC) ^a^	Phosphorylation (S210)	[22]
Protein farnesyltransferase (FTase) ^a^	Isoprenylation with farnesyl (C211)	[25–28]
Protein arginine methyltransferase 1 (PRMT1)	Methylation (R13)	[29]
p300 cellular acetyltransferase	Acetylation (K72)	[30,31]
Small ubiquitin-related modifier isoform 1 (SUMO1) ^b^	Sumoylation of multiple lysine residues	[32]
Ubc9^b^	Sumoylation of multiple lysine residues	[32]

	**Sub-cellular localization**	
karyopherin (importin) 2α	Nuclear import	[33]
Nuclear export signal-interacting protein (NESI)^a^	Nuclear export	[34]
Clathrin heavy chain^a^	Exocytosis	[35,36]
Nucleolin (C23)	Nucleolar localization, shuttling, RNA synthesis/accumulation (?)	[37]
Nucleophosmin (B23)	Nucleolar localization, shuttling, RNA synthesis/accumulation (?)	[38]

	**RNA synthesis**	
RNAP II (Rpb1/2 mobile clamp element)	Genome and mRNA synthesis Antigenome synthesis (?)	[39,40]
DRB sensitivity-inducing factor (DSIF)	Relieves transcriptional repression; stimulates elongation by RNAP II	[12]
*delta* interacting protein A	Transcriptional regulation (?)	[41]
Yin Yang 1 (YY1)	RNA synthesis/accumulation (?)	[30]
Histone H1e^b^	RNA synthesis/accumulation (?)	[42]

	**Other**	
MOV10	RNA remodelling (?)	[43]
Smad3	Alters host gene expression	[44]
c-Jun	Alters host gene expression	[44]
TRAF2	Alters host gene expression	[45]

(a) HDAg-L only.

(b) HDAg-S only.

**Table 2.** Host proteins that interact with HDV RNA.

**Host protein**	**Proposed Function**	**References**
Adenosine deaminase acting on RNA (ADAR 1)	Post-transcriptional modification of the antigenome that results in production of HDAg-L	[15]
Glyceraldehydes 3-phosphate dehydrogenase (GAPDH)	Enhances *delta* ribozyme activity	[58,59]
Double-stranded RNA-activated protein kinase R (PKR)	Recruitment to HDAg for post-translational modification Repression of antiviral response	[60,61]
RNAP I	Antigenome synthesis	[62]
RNAP II	Genome synthesis, mRNA synthesis, Antigenome synthesis	[63–66]
RNAP III	Unknown	[62]
Polypyrimidine tract-binding protein associated splicing factor (PSF)	Suspected involvement in recruitment of HDV RNA to RNAP II	[67]
54 kDa nuclear RNA-binding protein (p54^nrb^)	Unknown	[59]
Heterogeneous nuclear ribonucleoprotein L (hnRNPL)	Unknown	[59]
Arginine/serine-rich splicing factor (ASF)	Unknown	[59]
Eukaryotic elongation factor 1A1 (eEF1A1)	Unknown	[59]
